# Levels of CD56+TIM-3- Effector CD8 T Cells Distinguish HIV Natural Virus Suppressors from Patients Receiving Antiretroviral Therapy

**DOI:** 10.1371/journal.pone.0088884

**Published:** 2014-02-10

**Authors:** Bhawna Poonia, C. David Pauza

**Affiliations:** Institute of Human Virology, School of Medicine, University of Maryland, Baltimore, Maryland, United States of America; New York University, United States of America

## Abstract

Prolonged antiretroviral therapy (ART) with effective HIV suppression and reconstitution of CD4 T cells, fails to restore CD8 T cell lytic effector function that is needed to eradicate the viral reservoir. Better understanding of the phenotype and function of circulating CD8 cells in HIV patients will contribute to new targeted therapies directed at increasing CD8 T cell lytic effector function and destruction of the viral reservoir. We show that CD8 T cells from ART treated patients had sharply reduced expression of CD56 (neural cell adhesion molecule-1), a marker associated with cytolytic function whereas elite patients who control HIV in the absence of ART had CD56+ CD8 T cell levels similar to uninfected controls. The CD56+ CD8 T cells had higher perforin upregulation as well as degranulation following stimulation with HIV gag peptides compared with CD56 negative CD8 T cells. Elite patients had the highest frequencies of perforin producing CD56+ CD8 T cells among all HIV+ groups. In patients receiving ART we noted high levels of the exhaustion marker TIM-3 on CD56+ CD8 T cells, implying that defective effector function was related to immune exhaustion. CD56+ CD8 T cells from elite or treated HIV patients responded to PMA plus ionomycin stimulation, and expressed transcription factors T-bet and EOMES at levels similar to uninfected controls. Consequently, the lytic effector defect in chronic HIV disease is due to immune exhaustion and quantitative loss of CD56+ CD8 T cells and this defect is not repaired in patients where viremia is suppressed and CD4 T cells are recovered after ART.

Reconstituting the cytotoxic CD56+ subset of CD8+ T cells through new interventions might improve the lytic effector capacity and contribute to reducing the viral reservoir. Our initial studies indicate that IL-15 treatment partly reverses the CD56 defect, implying that myeloid cell defects could be targeted for immune therapy during chronic HIV disease.

## Introduction

The pronounced effects of HIV disease on cell subsets that are not permissive for HIV infection (indirect effects) broadens and deepens the immune system deficit. Among these indirect effects is a sharp reduction in cytotoxic potential of CD8 T cells [1] that is associated with defective activation due to modifications in the T cell receptor complex [2]. The CD8 T cell defects result in lower perforin and granzyme accumulation [3] and reduce the capacity for cytotoxic killing of cells infected by HIV or other pathogens. Functional defects were much less apparent among elite controller patients, who suppress HIV replication in the absence of therapy. These rare HIV patients retain a capacity to control new virus variants [4] and are less susceptible to apoptotic cell death [5]. The loss of cytotoxic effector function among CD8 T cells from most patients may explain why HIV persists for many years even when viremia is suppressed by effective therapy, but rebounds quickly when treatment stops. Our goals are to understand the mechanisms for indirect effects of HIV on CD8 T cells, define cell surface markers useful for measuring this immune defect and explore strategies to mitigate the CTL deficit in patients with HIV.

Fortunately, it is possible to identify cytotoxic effector CD8 T cells by expression of the CD56 cell surface glycoprotein. CD56 or neural cell adhesion molecule-1 (NCAM-1), is present on immune effector cells including NK and T lymphocytes. CD56 expression identifies cytotoxic subsets of NK, CD8 T and γδ T cells [6], [7], [8] but whether it has a functional role in cell killing remains unknown. The presence of CD56 identified the subset of CD8 T cells most potent for cytotoxicity [8], [9], [10], likely due to higher cellular content of perforin and granzyme B. CD56 expressing cells also contain higher levels of the anti-apoptotic protein Bcl-2 [9] and are mostly long-lived, effector memory.

What we know about the relationship of HIV infection to CD56 effector lymphocytes derives mainly from studies on NK and γδ T cells [11], [12]. Among NK cells, the cytotoxic CD56^low^CD16^hi^ subset is proportionally reduced in HIV patients, due to proliferation of CD16+CD56- cells [13], [14], [15], [16]. A benefit of antiretroviral therapy (ART) is to increase the proportion of cytotoxic CD56^+^ NK cells [15], [16], [17]. The proportion of CD56+ Vγ2Vδ2 T cells drops dramatically in HIV disease and does not return to normal levels even after prolonged ART [12], even where there is clear evidence for reconstitution of the Vγ2 chain repertoire by new cell synthesis [18]. Elite patients unique in their capacity to suppress viremia in the absence of therapy, have CD56+ Vγ2Vδ2 T cell levels and function similar to uninfected controls [12]. Untreated HIV infection reduces the levels of CD56+ CD8 T cells [19]; whether ART restores this subset is not known. The mechanism for HIV depletion of CD56+ lymphocytes is not known. The loss of CD56+ CD8 or γδ T cells may be due to indirect effects including bystander killing, transient modulation of CD56 expression or exhaustion of cytotoxic CD56+ cells.

In this study, we defined the characteristics of CD8 T cells in HIV+ or control subjects, emphasizing markers associated with lytic effector activity and immune exhaustion. CD8 T cell phenotypes were compared among patient groups including HIV-infected individuals receiving antiretroviral therapy (ART), patients with chronic low viremia in the absence of therapy (VIR), patients controlling virus replication in absence of therapy (natural virus suppressors (NVS) or elite controllers) and HIV-uninfected controls. These studies highlight a defect of CD8 T cells that is not reversed by long-term antiretroviral therapy and may be important for understanding durability of the latent viral reservoir.

## Methods

### Study subjects

We collected peripheral blood samples from three HIV infected and one uninfected (control) groups. A group of 20 HIV elite controllers (termed Natural Virus Suppressors, NVS) was selected randomly from a cohort defined previously [12]. NVS were naive to antiretroviral therapy. A group of 20 HIV-infected patients receiving ART (hereafter, HIV+ART) patients, all who had reconstituted CD4 after treatment initiation, represented the cohort defined previously [20]. The third group included 10 HIV-infected patients with low level viremia in the absence of therapy; they are designated chronic low viremia (VIR) hereafter [21]. The last group is of 20 HIV negative controls [20]. The clinical and demographic characteristics of study subjects are provided in Table 1.

**Table 1:** Demographic and Clinical Characteristics of Patients and Control groups.

Group (n)	Control (20)	Treated (20)	NVS (20)	VIR (10)
Characteristic	aHIV uninfected	bHIV infected treated with ART	bHIV infected, untreated, elite controllers	bHIV infected, untreated, chronic low viremia
Age, years,mean±SD	43.63±12.5	46.7±6.0	49.7±8.5	45.47±6.5
Male sex, %	40	68	67	48
CD4 cell count/ul, mean±SD	Not done	512±144	848±301	705±256

a HIV uninfected samples are blood from commercial source

b Data are for patients who received antiretrovirals, patients with natural virus suppression (NVS) and chronic low viremia (VIR) and are from [50].

### Ethics statement

Written informed consent was obtained from all patients or control subjects; the Institutional Review Board at the University of Maryland, Baltimore School of Medicine approved these protocols.

### Antibody staining and flow cytometry

Peripheral blood mononuclear cells (PBMC) were purified from venous blood, stored as viable frozen cells and used for immunophenotyping experiments. Cells (5×10^5^) were labeled with appropriate antibodies at 4°C for 30 min. The following monoclonal antibodies (from BD Biosciences, San Diego, California unless specified otherwise) were used in this procedure: anti-CD8 FITC (clone RPA T8), anti-CD16 FITC (clone 3G8), anti-CD3 PerCP (clone SP34-2), anti-CD3 PerCPCy5.5 (clone UCHT1), anti-CD56 APC (clone B159), anti-TIM-3 PE (clone F38-2e2, Biolegend, San Diego, CA), anti-CD107a FITC (clone H4A3) and the appropriate isotype controls. After staining, cells were washed once with phosphate-buffered saline (PBS) and fixed with 1% paraformaldehyde.

For intracellular perforin and granzyme B staining, cells were fixed after staining for surface markers, then permeabilized using the BD Cytofix/Cytoperm™ Fixation/Permeabilization Solution Kit and finally stained with anti-perforin (clone dG9, Biolegend) or anti-GranzymeB (clone GB11, Invitrogen) antibodies. Data from at least 2×10^4^ cells in the lymphocyte gate were collected on a FACSCalibur flow cytometer (BD Biosciences). Flow cytometry data were analyzed using FlowJo software (Tree Star, San Carlos, California, USA).

For analysis of the transcription factors EOMES or T-bet, cells were stained for surface markers followed by intracellular staining using anti-T-bet Brilliant Violet 421 (clone 4B10) and anti-EOMES e-Fluor 660 (clone WD1928). Flow cytometry data were collected on a FACS Aria flow cytometer.

For cell stimulation, staining for phospho-Erk and degranulation experiments, frozen cells were thawed (1×10^6^/ml) and rested overnight (approximately 18 hours) in RPMI complete medium with 10% fetal bovine serum (FBS), 1% penicillin-streptomycin, 2 mM L-glutamine before each experiment.

### Cell stimulation and Phosflow flow cytometry

HIV-specific responses of CD8 T cells were tested by studying upregulation of perforin after stimulation with HIV gag peptides (complete set of HIV gag overlapping peptides from NIH AIDS Reagent Program). The anti-perforin antibody (clone B-D48; Biolegend) that measures perforin accumulation in response to antigen stimulation [22], was used here. Additionally, degranulation by CD8 T cells after stimulation was measured with anti-CD107a antibody staining.

Cells were stimulated for 6 hours with a combination of HIV gag peptides, co-stimulatory antibodies CD49d plus CD28 at 1 µg/ml final concentration (BD Biosciences; San Jose, California), and protein transport blockers Brefeldin A at 1 μg/ml (BD Biosciences; San Jose, California) plus Monensin at 1 μg/ml (BD Biosciences; San Jose, California). CD107a antibody was added for the entire period of stimulation in respective samples. At the end of each incubation, cells were stained with surface and intracellular markers using BD Cytofix/Cytoperm kit (BD Biosciences; San Jose, California) according to manufacturer's instructions.

In order to measure MAPK signaling in CD56+ CD8 T cells, rested PMBC were stained for surface markers then stimulated with PMA (100 ng/ml) (Sigma) plus Inomycin (1 ug/ml) (Sigma) for 15 minutes at 37°C, fixed in Cytofix buffer (BD Biosciences) for 15 minutes at 37°C, permeabilized in ice cold Perm Buffer II (BD Biosciences), stained with p-Erk PE antibody (clone 612566), and fixed with 1% paraformaldehyde. Data from at least 2×10^4^ cells in the lymphocyte gate per sample were collected on a FACSCalibur flow cytometer (BD Biosciences).

### IL-15 treatments

PBMC from HIV infected or control individuals were plated at 1×10^6^ cells/ml along with of 10 ng IL-15 (Thermo Scientific, Rockford, IL) and cultured for 12 days. Cells were stained with surface marker antibodies and analyzed by flow cytometry at the end of each culture.

### Statistical analysis

Statistical analyses were performed with the Prism GraphPad software (GraphPad Software, San Diego, CA). Mann-Whitney test for two groups; Kruskal-Wallis test followed by a Dunns test for multiple comparisons when comparing three or more groups were used.

## Results

### CD56-expressing CD8 T lymphocytes are depleted in HIV patients on therapy but not in natural virus suppressors

**Figure 1:**
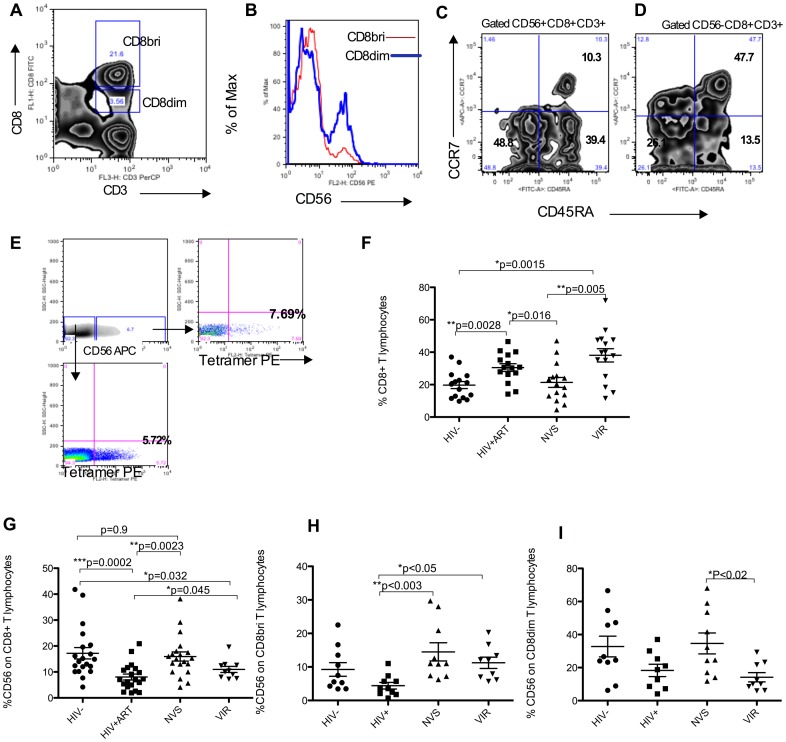
Preserved CD56+ CD8 T lymphocytes in elite controllers. (A, B) Higher expression of CD56 on CD8dim T compared with CD8bright T lymphocytes. (C) Representative samples shows CD56+ CD8 T lymphocytes are mostly effector memory type whereas (D) CD56- CD8 T lymphocytes have a significant subset of naïve and central memory and type cells. (E) Both CD56+ and CD56- subset of CD8 T cells have similar frequency of HIV tetramer binding cells. Cells from an HLAB5701 HIV infected individual were stained with TW10 tetramer. Cells are gated on CD8+ CD3+ lymphocytes. (F) Significantly elevated CD8 T cell frequencies in both ART treated and low viremia (VIR) groups but not in elite controllers (NVS). (G) The frequency of CD56+ CD8+ T lymphocytes is significantly reduced in HIV+ ART treated group compared with uninfected controls. NVS (elite controllers) did not show this defect and have normal levels of CD56 expression on CD8 T cells. (H) Analysis of CD8bri (H) or CD8dim (I) populations of T cells show that NVS group has superior levels of CD56 subset among both populations whereas ART group has lowest levels of CD8bri cells expressing CD56. VIR group has high frequency of CD56 expressing CD8bri but not CD8dim cells when compared with ART patients. For F-I, Kruksal-Wallis test followed by Dunn's test for multiple correction was used to analyze the four groups.

The cytotoxic potential of CD8 T cells is highest within a CD56-expressing subset [8], [9], [10]. Phenotyping studies showed that CD8 bright T lymphocytes express CD56 at lower levels compared with a CD8dim subset (Figures 1A, 1B), and CD56+ CD8 T cells are primarily of the effector memory/terminally differentiated (CCR7^−^CD45RA^−/+^) type compared to CD56- CD8 T cells that are mostly in the naïve (CCR7^+^CD45RA^+^) sub-population (Figure 1C, 1D). It is essential to identify the HIV-specific CD56+ CD8 T cells in order to understand their relevance for virus control. We obtained cells from two HIV+ elite controllers with the HLA*B57 haplotype. Using MHC Class I (TW10) tetramers loaded with gag peptides, we evaluated the phenotype of HIV-specific CD8 T cells. Tetramer staining revealed that similar proportions of both CD56+ and CD56- subsets of CD8 T cells were stained by the TW10 tetramer (Figure 1E) indicating that the HIV specific T cells are present in both sub-populations. This result is very similar to earlier studies on HPV-specific CD8 T cells where antigen-specific CD8 T cells appeared in CD56+ and CD56- sub-populations [10].

Next, we characterized the bulk CD8 T cell population because of previous reports [19] about CD56+ CD8^bri^ T cell depletion in untreated HIV patients. The frequency of CD8 T cells among all CD3+ cells was elevated in HIV patients receiving ART (HIV+ART) but not in NVS patients (Figure 1F). CD56 expression on CD8 T cells was significantly decreased in the HIV+ART group compared to uninfected controls or NVS patients (Figures 1G-I). Apparently, prolonged ART does not repair the CD56 defect even after CD4 T cell immune reconstitution. NVS showed high levels of CD56 on total or CD8^bri^ T cell population. The VIR group also had higher CD56 expression on CD8^bri^ or total CD8 T cells than did ART patients. We are reporting CD8 cell frequencies but similar conclusions were reached when absolute cell counts were used to analyze this subset in untreated HIV infection [19]. Thus patients who control virus replication without therapy preserve or recover the CD56+ CD8 cytotoxic T cell subset.

### Elite controllers have higher levels of perforin and Granzyme B in their CD56+ CD8 T cells

**Figure 2:**
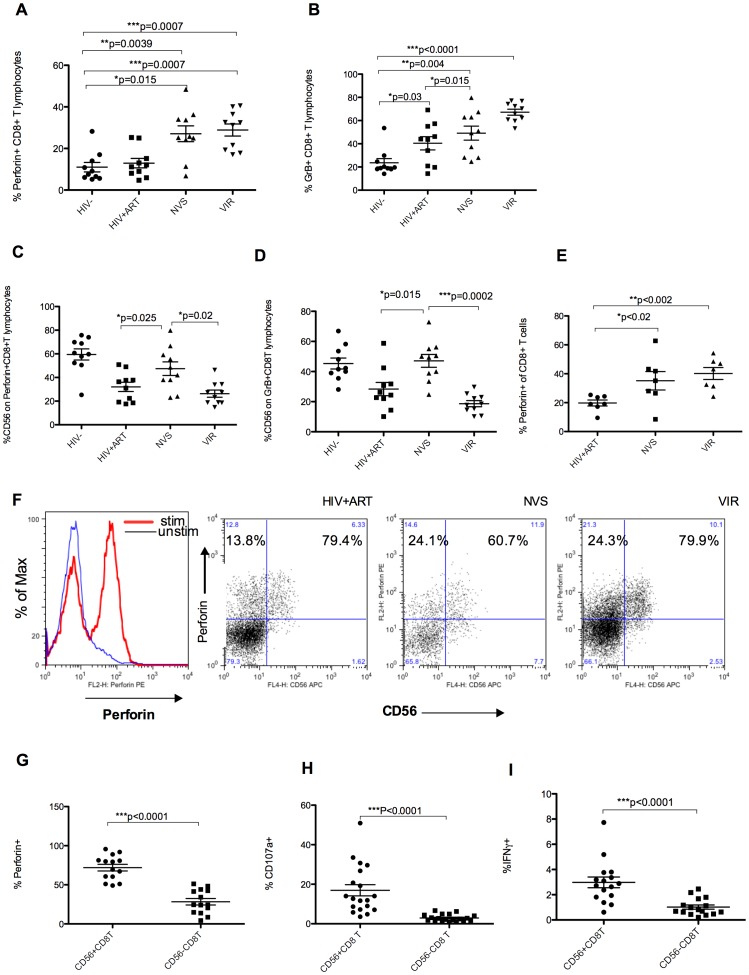
Elite controllers have higher levels of perforin/GranzymeB+CD56+ CD8 T lymphocytes. Intracellular expression of preformed perforin (A) and Granzyme B (B) is significantly higher on gated CD8 T lymphocytes from elite controllers and low viremia groups compared to uninfected controls or HIV+ART subjects. The level of CD56 expression on perforin (C) or Granzyme B (D) expressing CD8 T lymphocytes was significantly reduced in HIV+ ART treated and low viremia groups but not in elite controllers. Results in panels E-G show perforin upregulation in response to stimulation with HIV gag peptides measured with DG48 clone of anti-perforin antibody. (E) Both elite controller and low viremia group have significantly higher production of perforin upregulation in response to stimulation compared with ART treated individuals. (F). Representative sample from each infected group showing majority of CD56+ CD8 T cells upregulated perforin in response to stimulation while a minority of CD56- CD8 T cells upregulate perforin. Cells are gated on CD8 T lymphocytes. (G) Combined results from all infected groups show proportion of CD56 fraction of CD8 T cells upregulating perforin in response to stimulation with HIV peptides is significantly higher than proportion of CD56- CD8 T cell that upregulate perforin. Significantly greater proportions of CD56+ CD8 T cells degranulate (H) as well as produce IFNγ (I) in response to stimulation with HIV gag peptides. Results in G, H and I show show comparison of CD56+ and CD56- subsets irrespective of the patient's disease status.

Levels of preformed perforin or granzyme B are predictive of cytotoxic effector potential. In CD8 T cells from NVS or VIR groups, perforin and granzyme B were significantly above levels found in controls or HIV+ ART patients (Fig. 2 A, 2B). When we gated on the perforin+ or granzyme B+ CD8 T cells, CD56 expression was substantially higher for NVS compared with other HIV infected groups, and was similar to HIV-uninfected controls (Figures 2C, 2D). We can discriminate pre-formed from newly expressed perforin using the B-D48 clone of anti-perforin antibody. The appearance of newly-formed perforin is a correlate of virus control [22]. NVS and VIR groups had significantly higher levels of perforin upregulation in response to stimulus (Figure 2E), indicating higher proportions of antigen-specific cells with cytotoxic potential. This corresponds with the high frequency of CD8^bri^ CD56+ T cells in these groups. As shown in representative examples (Figure 2F), a majority of CD56+ CD8 T cells from all infected groups, upregulated perforin in response to stimulation compared with much lower perforin response in the CD56- subset (p<0.0001) (Figure 2G). While there were no significant differences in the overall levels of degranulation comparing CD8 T cells from groups of infected patients (data not shown), CD107a surface expression increased to a greater degree in CD56+ compared to the CD56- cells (Figure 2H). Similarly, IFNγ responses to HIV gag peptides was significantly (p<0.0001) higher in the CD56+ CD8 T cell subset than in CD56- cells (Figure 2I).

### CD56+ CD8 T cells in HIV infection co-express T-bet and EOMES and have intact signaling

**Figure 3:**
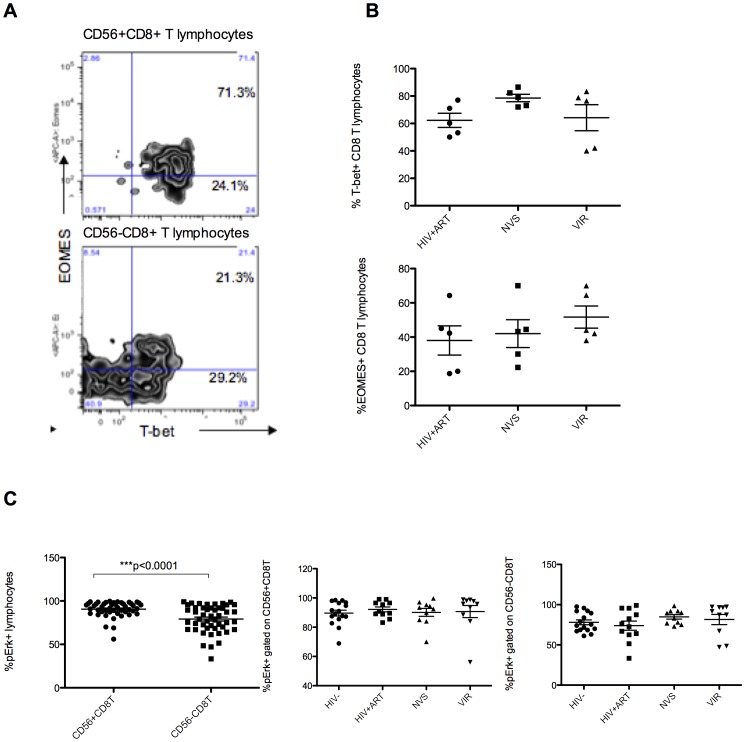
Transcription factors and MAPK signaling in CD56 subset. EOMES and T-bet control cytotoxic function of effector T cells. (A) Majority of CD56+ CD8 T cells expressed T-bet and/or EOMES and were double positive for these transcription factors, whereas a significant proportion of CD56- CD8 T cells were double negative for EOMES and T-bet. The representative plots show the trends presend in CD56+ or CD56- subsets from all patient groups irrespective of disease status (B) NVS group had highest frequency of T-bet expressing CD8 T cells whereas there was no significant difference in EOMES positive CD8 T cells among different cohorts of HIV infected patients. (C) Overall, the CD56+ fraction of CD8 T lymphocytes had higher levels of Erk phosphoryation upon PMA+inomycin stimulation compared with the CD56- CD8 T lymphocytes when samples from all groups were combined for analysis. CD56+ or CD56- CD8 T lymphocytes showed similar levels of Erk phosphorylation in all infected and control groups.

We are trying to understand influence of HIV infection on levels and function of CD56+CD8 T cells. We looked at expression of major transcription factors related to cytotoxic effector function that are also disrupted by HIV infection and found that CD56+ CD8 T cells have significantly higher levels of T-bet and EOMES compared with CD56- cells. The CD56+ CD8 T cells expressing T-bet mostly co-expressed EOMES (Figure 3A). In contrast, a large proportion of CD56- cells were negative for both T-bet and EOMES (Figure 3A), a phenotype predicted to have reduced lytic effector function [23], [24]. Recently, HIV infection was shown to downregulate both of these transcription factors in CD8 T cells [23]. There were no significant differences in T-bet or EOMES levels for CD56+ subsets from patient infected groups although we noted that NVS had higher proportions of T-bet expressing CD8 T cells (Figure 3B), similar to earlier reports that HIV specific CD8 T cells from elite controllers expressed T-bet [25]. We did not find differences in the frequency of EOMES expressing CD8 T cells among any of the infected groups (Figure 3B).

HIV infection interferes with the MAPK signaling pathway and patients with uncontrolled infection accumulate a population of CD8 T cells refractory to Erk phosphorylation that associate with poorer virologic control [26], [27]. We tested whether the CD56+ subset of CD8 T cells in HIV patients is defective for MAPK signaling. HIV+ and control groups were compared in terms of sensitivity to PMA+I stimulation. Overall, the frequency of p-Erk1/2+ cells was significantly higher in the CD56+ subset compared with CD56- subset for all groups with no significant differences among infected or control groups (Fig. 3C). These results show that CD56+ CD8 T cells have an exceptional ability to signal through Erk and while patients on ART have numerical defect in CD56 expressing CD8 T cells, they have normal response to stimulus.

### High expression of the exhaustion marker TIM-3 on of CD56+ CD8 T cells from HIV patients

**Figure 4:**
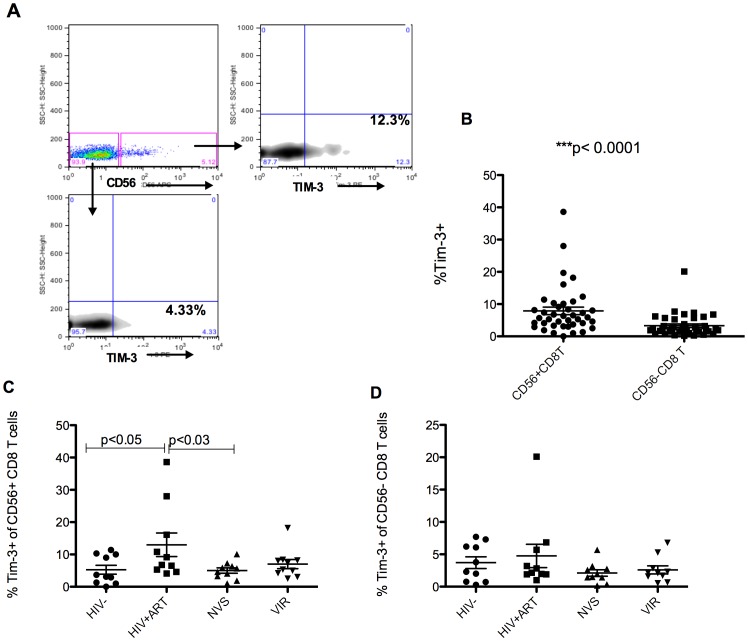
Exhausted CD56+ CD8 T lymphocytes in treated HIV infection. The expression of Tim-3 on T cells identified exhausted T lymphocytes. (A) A higher proportion of CD56+ CD8 T cells express Tim-3 compared to the CD56- subset. (B) Cumulative data from all individuals irrespective of disease status shows significantly higher Tim-3 expression by CD56+ CD8 T cells (C) The proportion of CD56+CD8+ T cells that are exhausted as judged by their Tim-3 expression were significantly higher in HIV+ART but not in elite controllers when compared to uninfected group. (D) The levels of Tim-3 on CD56- CD8 T cells is comparable in all HIV- and HIV+ groups.

HIV infection leads to accumulation of effector T cells characterized by enhanced expression of TIM-3 [28], a marker for immune exhaustion. TIM-3 is expressed on terminally differentiated TH1 cells and triggers cell death after binding its ligand Galectin-9 [29]. Since a majority of CD56+ CD8 T cells are terminally differentiated effector memory (CD45RA+CCR7-), upregulation of TIM-3 might explain how CD56+ CD8 T cells are lost during HIV disease. The levels of TIM-3 were higher in the CD56+ versus CD56- subset of CD8 T cells in controls or patients (Figure 4A, 4B). When analyzed according to disease status, among the CD56+ subset, the NVS group had TIM-3 levels similar to uninfected controls whereas HIV+ ART group had elevated TIM-3 on CD56+ subset (Figure 4C). TIM-3 was not significantly different on CD56- CD8 T cells in any group suggesting that the CD56+ subset is exhausted and depleted in HIV infection by this mechanism.

### IL-15 upregulates CD56 expression on CD8 T cells from HIV infected patients

**Figure 5:**
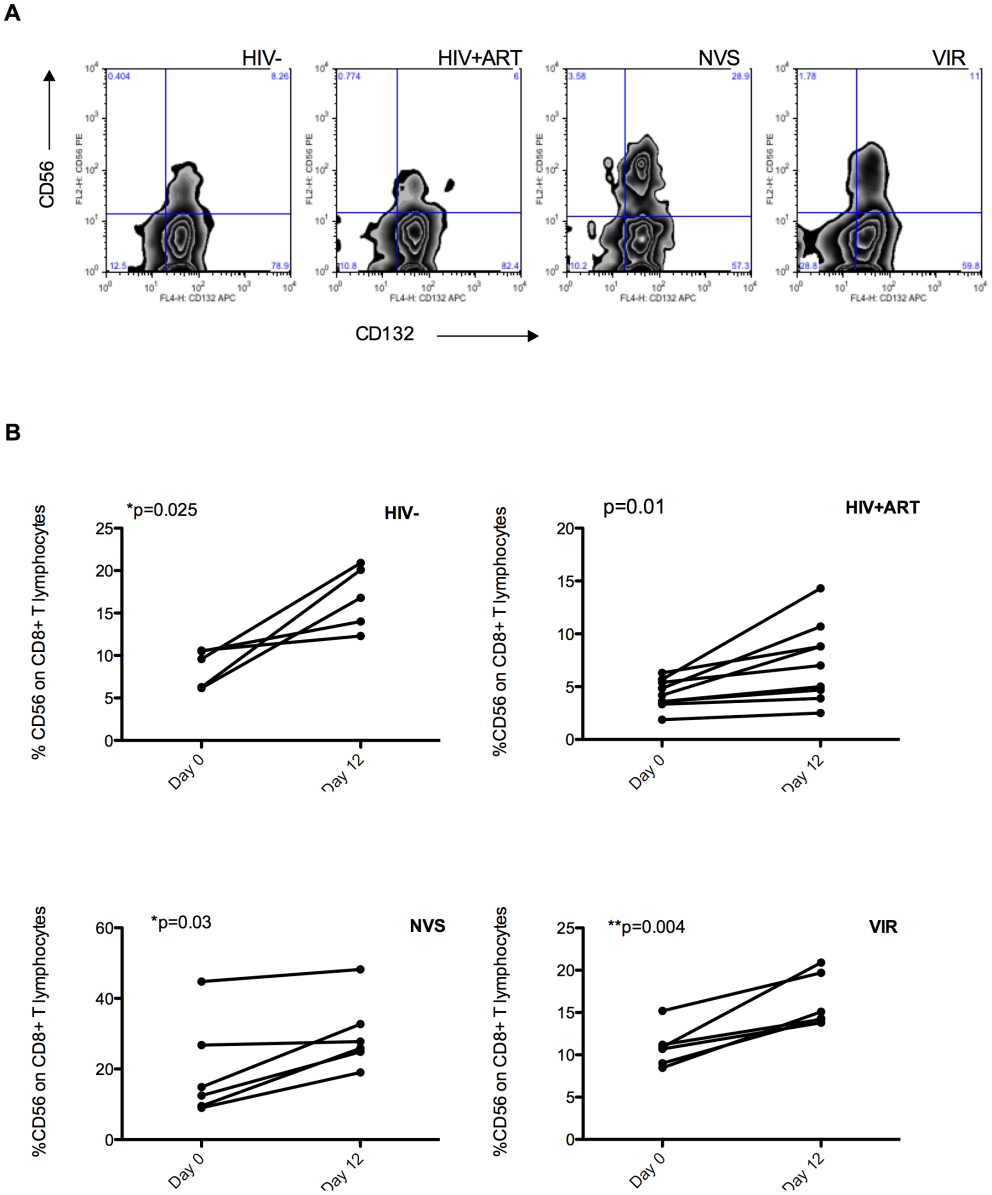
IL-15 restores CD56 on CD8 T cells from HIV infected patients. (A) Most CD8 T cells and almost all CD56 + CD8 T cells express the common gamma chain (γ_c_) (or CD132). Representative plots (from left ND, PD, NVS, VIR) show there was no significant downregulation of this receptor on CD8 T lymphocytes from any infected group (B) Culture of PMBC in IL-15 resulted in a significant upregulation of CD56 on CD8 T lymphocytes at day 12 relative to start of culture in infected and uninfected samples.

One of our long-term goals is to restore CD56 expression on CD8 T cells from HIV patients and hopefully reconstitute effector function. Common γc cytokines like IL-2, IL-7, IL-15 and IL-21 are important for CD8 T cell homeostasis and generating memory; they are candidates for restoring lytic effector function. Among them, IL-15 was most effective for upregulating CD56 on CD8 T cells (data not shown). However, HIV infection down regulates the common γc (CD132) on CD8 T cells in acute infection [30] and this could make cells unresponsive to common γ cytokines. CD132 was present at similar levels in all groups in this study (Figure 5A) and adding IL-15 increased CD56 expression CD8 T cells (Figure 5B). It will be critical to study if such IL-15 expanded CD56+ CD8 T cells are functional effectors. We have not yet done cytotoxicity assays due to low cell numbers in our repository specimens, however, in HIV-negative subjects IL-15 treatment increased functional cytotoxic CD56+ CD8 T cells [9]. We showed above that the remaining CD56+ CD8 T cells from ART patients have normal response to stimulus including perforin upregulation, degranulation and MAPK signaling and thus we expect CD56+ CD8 T cells generated by IL-15 treatment of HIV patient samples to be functional cytotoxic effectors.

## Discussion

Prolonged antiretroviral therapy (ART) with effective HIV suppression leads, in most cases, to reconstitution of CD4 T cells [31]. Because *de novo* cell synthesis is expected to produce all T cell lineages (γδ, CD4 and CD8) patients reconstituting CD4 T cells are expected to have newly synthesized CD8 T cells as well. However, cytotoxic effector capacity remains low even after years of successful antiretroviral therapy. The failure to recover cytolytic effector function in CD8 T cells [3], [32], [33], [34], [35] is a critical defect that partly explains the inability to eliminate the reservoir of infected cells. If we can learn to reconstitute cytotoxic effector function, we may come closer to replicating the elite controller status for a greater proportion of patients with HIV disease.

CD8 T cell effectors in HIV control are defined by their surface markers, activation status, responses to stimulus, and functional profiles in elite patients [26], [36], [37]. We have focused most on the surface glycoprotein CD56, which defines the potently cytotoxic subpopulation [8], [9]. We showed that CD56+ CD8 T cells are enriched for effector memory, perforin/granzyme activity and transcription factors T-bet and EOMES. We found highest expression of CD56 on a CD8^dim^ subset. Previously, CD8^lo^ T cells were shown to have higher non-cytolytic HIV suppressing activity [38] and loss of this activity would further weaken host defense against HIV. CD56 expressing cells among the CD8^bri^ subset are likely to be the cytotoxic cells that are present at highest levels in the NVS group but depleted in ART patients. Epitope-specific CD8 T cell responses include both CD56+ and CD56-negative subpopulations and indeed, we found similar frequencies of HIV-specific, tetramer-binding cells among CD56+ and CD56- subsets of CD8 T cells from elite patients. Against HPV infected tumor cells, the CD56+ subset was much more cytotoxic than CD56- cells despite high levels of HPV specific CD56- CD8 T cells [10]. We know that only a fraction of HIV tetramer positive cells produce perforin [35], [39] and this reinforces a view that epitope specificity is not a marker for cytolytic effector function in CD8 T cells. We believe that the tetramer binding CD56+ CD8 T cell subset defines the most potent, virus-specific lytic effectors. This relationship between CD56 expression and cytotoxicity in CD8 T cells [8], [9], [10] is also seen with γδ T cells [6]. For the majority of CD56+, but not CD56- CD8 cells, perforin formation and degranulation increased after stimulation with HIV gag peptides, reinforcing the relationship between CD56 and lytic effector activity. HIV-specific CD8 T cells from elite controllers have been shown to have superior capacity to upregulate perforin which would help to eliminate infected cells and suppress viremia without therapy [22], [40]. Besides cytotoxicity, CD56+ cells also have non-cytolytic anti-HIV activity via CC-chemokines as was shown [41], and preservation of this subset in elite controllers but not in ART treated patients represents a basic difference in capacity for immune control of viremia.

Surprisingly, the role for CD56 in lytic effector activity remains unknown. CD56 is a poly-sialylated cell surface glycoprotein that directs neural cell aggregation *in vitro* [36], [42]. In earlier studies, we tested antibody blocking of CD56 or removal of poly-sialic acid chains on CD56+ γδ T cells. These treatments tended to reduce cell proliferation or lower lytic effector activity, but none of these data were convincing enough to publish. In our hands and in the literature [37], we still don't know whether CD56 expression on effector cells is incidental or required for cell killing. Our results show that CD56 expression on CD8 T cells is a correlate of both progressive HIV infection and elite control. Thus, this phenotypic marker should be used to monitor recovery of anti-HIV potential of CD8 T cells from patients undergoing ART.

We also observed that NVS group had higher expression of CD56 on total CD3+ T cells compared to HIV+ ART individuals (data not shown). The same NVS cohort had higher expression of CD56 on γδ T cells compared to HIV+ ART patients [12], suggesting a selective preservation of cytotoxic T lymphocyte subsets in NVS patients. Whether these patients experienced an early depletion of CD56+ T cells and later recovered these cells is not known. Interestingly, NK cells defined by CD56 were not elevated in elite patients and had similar phenotypes among all infected groups (data not shown). Understanding how and why CD56+ CD8 T cells are lost in HIV disease may reveal critical mechanisms of pathogenesis. During chronic disease, the lytic effector capacity is reduced sharply [43] and in treated patients, the decline in HIV-specific effector activity is often attributed to lower antigenemia. However, elite patients do not show similar defects in spite of very low antigen levels. Such comparisons argue for active suppression or depletion of CD56+ CD8 T cells even in patients where viremia is suppressed during years of successful ART.

We tested whether immune exhaustion of CD8 T cells can explain their loss in HIV+ patients. Considering recent reports on the use of PD-1 as a marker [44] and our own studies showing little variation in PD-1 levels across control and HIV+ groups (unpublished), we measured TIM-3 expression as a surrogate for immune exhaustion [28], [45]. Among elite patients, few of the CD56+ CD8 T cells were positive for TIM-3. The proportion of TIM-3+ CD56+ CD8 T cells was significantly higher for HIV patients receiving ART, making immune exhaustion a potential mechanism for preferential depletion of CD56+ CD8 T cells. Besides immune exhaustion, CD8 T lymphocytes from HIV infected individuals undergo spontaneous apoptosis [46] seemingly as a result of growth factor deprivation. Exogenous IL-15 is a potent survival factor for CD8 T cells, preventing their apoptosis by increasing the expression of anti-apoptotic Bcl-2 [46]. We showed here that CD56 expressing CD8 T lymphocytes from HIV infected individuals were significantly expanded *in vitro* by IL-15, indicating that a similar phenomenon may be responsible for their apoptosis during chronic infection. IL-15 is known to upregulate Bcl-2 in CD56+ CD8 T cells and increase their survival [9]. Total and HIV-specific CD8 T cells from elite controllers have increased levels of Bcl-2 [5] and this may preserve the CD56+ subset of CD8 T cells. Our results also raise important questions about the mechanism for immune reconstitution in patients receiving ART. It is believed by several groups, that the potential to express CD56 and become a cytotoxic effector cell, is acquired at or near the time of thymic differentiation [47], [48]. If CD4 reconstitution is due to new thymic output as claimed by several groups [49], [50], we would expect reconstitution of all T lymphocyte subsets including CD8 T cells, with recovery of the CD56+ subpopulations. That the CD56+ subpopulations are not recovered in γδ [12] or CD8 T cells after prolonged ART with CD4 reconstitution, suggests a pathogenic process able to delete CD56+ cells is ongoing even with potent virus suppression due to antiretroviral therapy. Understanding how and why CD56+ cells are lost in HIV disease may reveal critical disease mechanisms and new diagnostic or therapy targets. In summary, the results presented here show that HIV elite controllers preserve the highly functional CD56 expressing CD8 effector T cells, in contrast to individuals in which HIV replication is suppressed by ART. Inclusion of CD56 marker in flow cytometric studies might be useful to monitor levels of cytotoxic CD8 T cells in HIV infection. The levels of CD56 expressing CD8 T cell subset correlate with elite controller status. One approach to replicating the elite status for all HIV patients, is to devise strategies to replenish the CD56+ lytic effector subset of CD8 T cells.
